# *Spigelia genuflexa* (Loganiaceae), a new geocarpic species from the Atlantic forest of northeastern Bahia, Brazil

**DOI:** 10.3897/phytokeys.6.1654

**Published:** 2011-09-14

**Authors:** Alex V. Popovkin, Katherine G. Mathews, José Carlos Mendes Santos, M. Carmen Molina, Lena Struwe

**Affiliations:** 1 Fazenda Rio do Negro, Entre Rios, Bahia, Brazil; 2 Department of Biology, 132 Natural Science Building, Western Carolina University, Cullowhee, NC 28723, USA; 3 Área de Biodiversidad y Conservación, Departamento de Biología y Geología, ESCET, URJC Móstoles, 28939 Madrid, Spain; 4 Dept. of Ecology, Evolution, & Natural Resources, Rutgers University, 14 College Farm Road, New Brunswick, NJ 08901, USA; 5 Dept. of Plant Biology and Pathology, Rutgers University, 59 Dudley Road, New Brunswick, NJ 08901, USA

**Keywords:** Dwarfism, evolution, geocarpy, ITS, Loganiaceae, Neotropics, phylogeny, Spigelieae

## Abstract

A new species of *Spigelia* L. (Loganiaceae), *Spigelia genuflexa* Popovkin & Struwe, **sp. n.**, from the Atlantic forest of northeastern Bahia, Brazil, is described, being the first reported geocarpic species in the family. During fruit maturation, the basal infructescences bend down towards the ground, depositing the fruit on the surface (and burying it in soft kinds of ground cover, e.g., moss), whereas the upper ones do so slightly but noticeably. The species is a short-lived annual apparently restricted to sandy-soil habitat of the Atlantic forest of northeastern Bahia, with variable and heterogeneous microenvironment and is known from only two restricted localities. A short review of amphi- and geocarpic species is provided. A discussion of comparative morphology within *Spigelia* with regards to dwarfism, indumentum, and annual habit is included. A phylogenetic parsimony and Bayesian analysis of ITS sequences from 15 *Spigelia* species plus 17 outgroups in Loganiaceae confirms its independent taxonomic status: on the basis of sequence similarity and phylogenetic topology it is phylogenetically distinct from all *Spigelia* species sequenced so far.

## Abstract (es)

Uma nova espécie de *Spigelia* L. (Loganiaceae), *Spigelia genuflexa* Popovkin & Struwe, **sp. n.**, da Mata Atlântica do litoral norte da Bahia, Brasil, é descrita, sendo o primeiro registro de espécie geocárpica na família. Durante a maturação do fruto, as inflorescências basais inclinam-se em direção ao solo, depositando os frutos sobre a superfície (ou mesmo enterrando os frutos sob a cobertura não compactada, e.g. musgos), enquanto as superiores inclinam-se apenas ligeiramente, mas de forma bem perceptível. A espécie é anual, aparentemente restrita à habitat sobre solo arenoso da Mata Atlântica do litoral norte da Bahia, com microambiente variável e heterogêneo, e é conhecida por apenas duas localidades. Uma breve revisão de espécies amfi- ou geocárpicas é apresentada. Também é feita uma discussão comparativa da morfologia em *Spigelia*, principalmente com relação à nanismo, indumento e hábito anual. Uma análise filogenética de parcimônia baseada em sequências de ITS de 15 espécies de *Spigelia* e de outras 17 espécies em Loganiaceae como grupo externo confirma o status taxonômico independente da nova espécie: a similaridade da sequência e a topologia da árvore filogenética demonstram que ela é filogeneticamente distinta de todas as outras espécies de *Spigelia* sequenciadas até agora.

## Introduction

*Spigelia* L. is a genus of approximately 60 species of Neotropical herbs to shrubs (Zappi 2005). It is distributed from temperate South America (about the latitude of Buenos Aires, Argentina) northward into the tropics of South America, to Central America, Mexico and the Caribbean, and into the warm-temperate southern United States. *Spigelia* species inhabit mid-elevation to lowland areas, with at least 60% of the species found in South America. Forty-three species are distributed in Brazil, including 15 in the state of Bahia, nine of which inhabit the Atlantic forest biome (“Mata Atlântica," Zappi et al. 2010) where the new species is found. There are also centers of diversity in the grassland regions of southern Paraguay and adjacent northeastern Argentina, as well as in the Mexican central highlands and wet lowland tropics of Mexico and Central America. Although most species are geographically restricted, several are widespread from North to South America, including *Spigelia anthelmia* L., *Spigelia humboldtiana* Cham. & Schltdl., and *Spigelia hamellioides* Kunth, with *Spigelia anthelmia* also naturalized in Africa and Malaysia.

Morphologically, *Spigelia* species can be recognized by their opposite or whorled leaves, one-sided cymose inflorescences, often brightly colored pentamerous flowers with usually funnelform or tubular corollas, articulated styles, and strongly bilobed capsules with persistent style and fruit bases.

The new species was discovered by José Carlos Mendes Santos (a.k.a. Louro), the house help and fellow plant collector of the first author, when squatting near the latter's house. The tiny plant of no more than 3 cm in height would have been otherwise easily missed. A colony of half-dozen plants, within 5 square meters, was initially discovered. Two more colonies in the same restricted area were eventually uncovered. The habitat is an open-soil roadside, partially covered by leaf litter, at the border of a *tabuleiro* forest in the Atlantic forest biome of northeastern Bahia, Brazil. The species has been observed for a period of over two years, during weekly visits. This is an ephemeral rainy-season species, with plants almost completely disappearing in the dry season. While collecting at a patch of the well preserved *tabuleiro* forest some 10 km east of the first find, additional, larger specimens (10–25 cm high) were discovered in forest border leaf litter by the same collectors (*Popovkin & Mendes 913*).

Relationships among the species of *Spigelia* are still poorly understood. Early phylogenetic results focusing on the north-temperate species showed that there are two distinct north-temperate lineages of *Spigelia* and that both of them have close relatives in the tropics (Gould 1997). *Spigelia* itself is an isolated lineage and forms a monotypic tribe in the Loganiaceae (Struwe et al. 1994; Frasier 1998). We obtained DNA sequence from the new species in order to confirm its membership in *Spigelia* and to see where it is positioned relative to other species in our working phylogenetic hypothesis for the genus.

## Taxonomic Treatment

### Spigelia genuflexa

Popovkin & Struwe sp. nov.

urn:lsid:ipni.org:names:77114017-1

http://species-id.net/wiki/Spigelia_genuflexa

Figs 1 2 

#### Additional photos at Popovkin: 

 http://calphotos.berkeley.edu/cgi/img_query?where-taxon=Spigelia+sp.+nov.&where-lifeform=specimen_tag&rel-lifeform=ne&rel-taxon=begins+with&title_tag=Spigelia+sp.+nov.; http://bit.ly/io7bpT--2009-2011

#### Diagnosis.

Haec species *Spigelia flemmingiana* Cham. & Schltdl. similis, sed plantis brevioribus (1.5–25.0 vs. 17–50 cm), foliis parvis (0.6–2 × 0.2–0.5 cm vs. 2–9 × 1.4–2 cm) ellipticis vel ovatis (vs. lanceolatis), corollis brevioribus (0.4–0.8 vs. ca. 1 cm), inflorescentiis paucifloribus, et infrutescentiis nutantibus in maturitatem (vs. semper erectis) differt.

Similar to *Spigelia flemmingiana* Cham. & Schltdl. but shorter (1.5–25 cm vs. 17–50 cm tall), with smaller leaves (0.6–2 × 0.2–0.5 cm vs. 2–9 × 1.4–2 cm) that are elliptic to ovate (vs. lanceolate), shorter corollas (0.4–0.8 cm vs. ca. 1 cm), fewer-flowered inflorescences (up to 7 flowers vs. up to 38 flowers), and infructescences bending downward at maturity (vs. staying erect).

#### Type.

** Brazil:** Bahia: Entre Rios, Fazenda Rio do Negro, Residual stands of the Atlantic Forest. Restinga-type forest of the Rio do Negro valley, ca. 15 km southeast of Entre Rios, Atlantic forest, 12°01'S, 38°02'W, 150 m, 31 July 2009, *A.V. Popovkin & J.C. Mendes 617* (holotype: HUEFS).

#### Description.

 Annual herb, 1.5–25 cm tall. Roots fibrous, not very extensive. Stem branched at base, with reddish tint, with 4–6 prominent ribs decurrent from the leaf bases; interpetiolar stipules triangular, with abundant papillae on outside. Leaves opposite as well as 4 together higher up on the main branch under the inflorescence, 6–20 mm long, 2–5 mm wide, elliptic to ovate; secondary veins 4–6 pairs, arcuate, inconspicuous below and above, midrib raised below; base acute, with decurrent lamina; margin flat or slightly revolute, entire; apex obtuse; upper side with many short, transparent papilloid hairs, 0.1–0.3 mm long; lower side glabrous; petiole 1–2 mm long. Inflorescence variable, solitary (occasionally multiple), typically a one-sided cyme (rarely a simple cyme/dichasium or a single flower), unbranched, (1-)4–7-flowered, up to 28 mm long, without bracts or with 1–2 tiny bracts subtending flowers; peduncle 7–15 mm. Flowers actinomorphic, perfect, 5- (rarely 6-) merous. Calyx divided almost to base, green, persistent in fruit; lobes triangular, acuminate, 0.8–1.4 mm long, c. 0.3 mm wide, with slightly papillose margins. Corolla sympetalous, tubular, slightly widening towards mouth, 4–8 mm long, 2.5–3.0 mm wide at mouth, white with pink lobes, aestivation valvate with individual corolla lobes plicate in bud, lobes unfolded when open, closing after a short (8-hour) anthesis, later withering and deciduous; lobes triangular, 1.0–1.5 mm long, ca. 1 mm wide, erect, acute, with smooth margin. Stamens epipetalous and adnate to corolla up to middle of the tube, of equal length, included in corolla; filaments flattened; anthers 0.7–0.8 mm long, shallowly sagittate at base, truncate at apex. Ovary bicarpellate, bilocular, ovoid, ca. 0.4 mm tall, with truncate apex; style 3–6 mm long (including stigma), simple, articulated at 0.5–1.00 mm above the ovary, mostly dehiscent in fruit (except the persistent base); stigma simple, papillose, ‘brush-like' at the height of the anthers. Fruit a bilobed capsule, 1.5–2 mm tall, 2–3 mm wide; dehiscing septicidally, loculicidally and circumscissilly, leaving behind on the rachis a persistent, boat-shaped base with pointed tips (‘*carpoatlas*' in Fernández Casas' (2003) terminology); light brown, warty to papillose; with ca. 0.5 mm tall style remnant. Seeds brown, round, reticulate surface when dry, ca. 0.7–1 mm in diameter.

**Figure 1.**
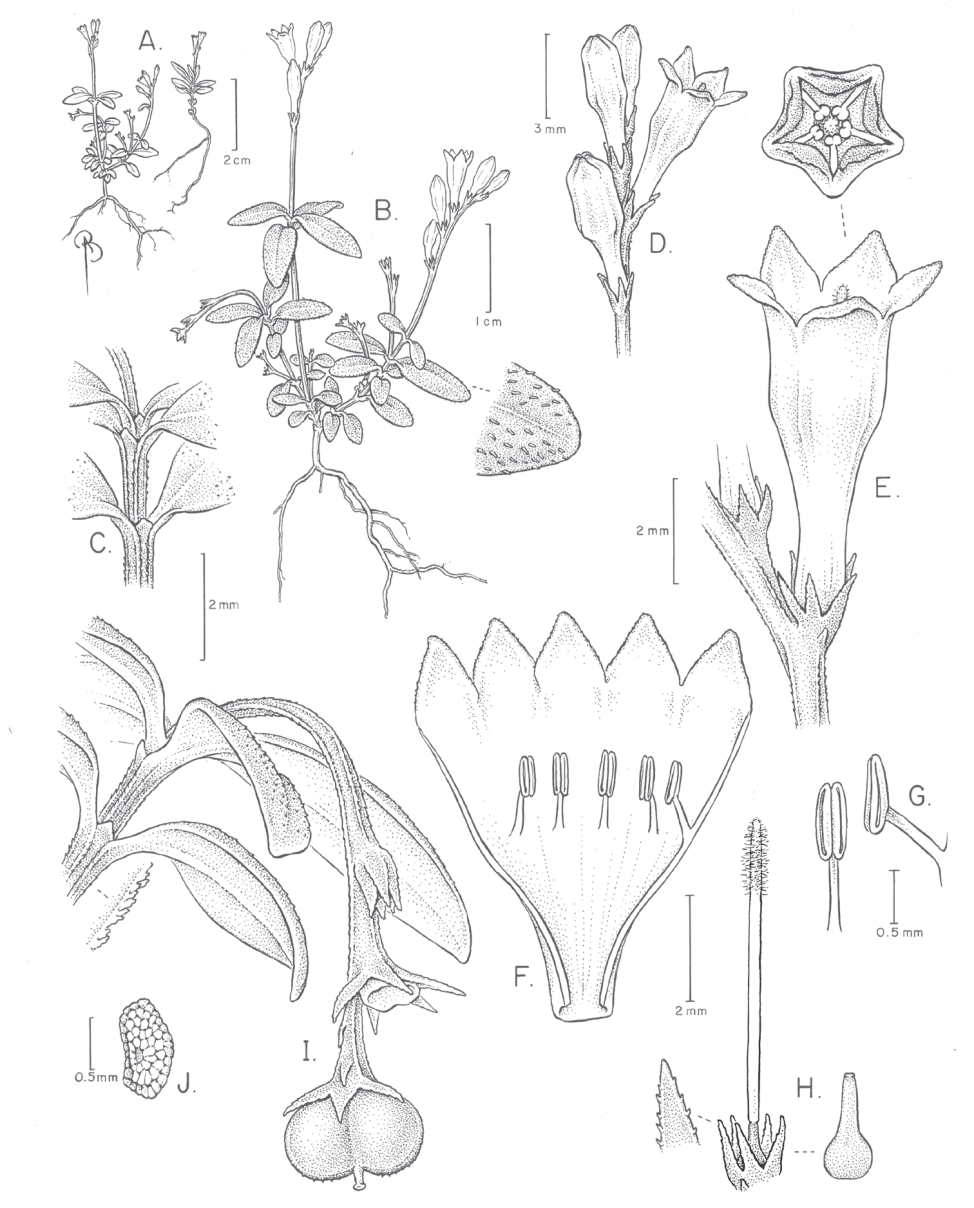
*Spigelia genuflexa* **A–B** Habit, showing inflorescences and geocarpic infructescenses, and close-up of apical part of leaf with apressed papilloid hairs **C** Close-up of node and internode, showing small triangular interpetiolar stipules **D** Flowers before and at anthesis **E** Close-up of flower at anthesis; note diminutive bract **F** Opened corolla with epipetalous stamens **G** Stamen inserted into corolla and introrse anther **H** Gynoecium inside papillose calyx, with hairy style (brush-type); older gynoecium, after style has dried and fallen off to the right **I** Geocarpic infructescence branch with one whole capsule (mitra-shaped, with small style remnant in center), and capsular base of the fruit that has dehisced, above it **J** Seed. Drawing by Bobbi Angell, based on *A.V. Popovkin 602 and 602A*.

**Figure 2.**
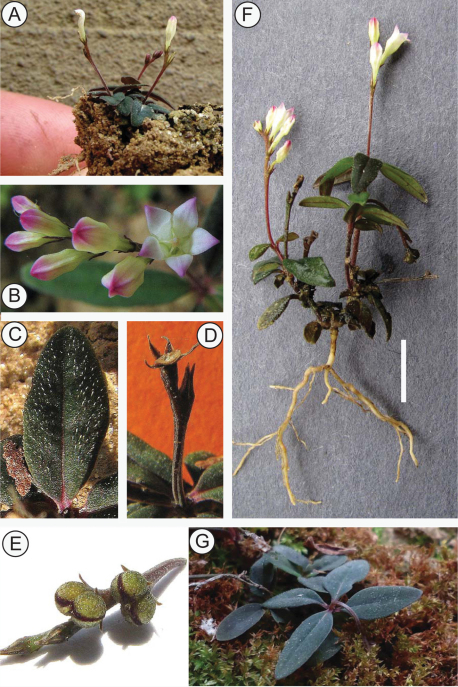
*Spigelia genuflexa* **A** Habit of mature plant **B** Flowers at anthesis and before opening. Note valvate and vertically folded petal lobes **C** Close-up of leaf with apressed papilloid hairs **D** Base of fruit after dehiscence (‘carpoatlas') **E** Fruit before dehiscence **F** Whole plant with roots. Scale bar = 1 cm **G** Infructescence showing geocarpy. Photos by Alex Popovkin.

#### Distribution.

 This species is known from only two localities in northeastern Bahia (Brazil), about 30 km from the Atlantic coastline.

#### Ecology.

 The species has been found on sandy,leaf litter- or moss-covered soil areas along the border of a *tabuleiro* forest. The diminutive flowers appear to be able to self, based on observations of cultivated material, with one to two flowers opening at one time. The anthesis begins early in the morning and ends in the afternoon of the same day. The arrangement and morphology of stamens and pistil, with anthers located closely to the central pistil with hairy upper part (Figure 1), suggests that spatial closeness of flower parts may promote selfing, thus ensuring fruit set. Occasional tiny ant visitors have been observed entering the open flowers, though it is not entirely clear if they might be the pollinators.

#### Dispersal.

 The geocarpy, i.e. weak geocarpy (depositors, in Hylander's [1929] terminology), of this species was initially observed on plants transplanted to a pot kept on a windowsill, allowing for daily/hourly observations. Two growth forms have been observed: one with inflorescences forming after the first three pairs of leaves are formed (usually, with a long internode between the first pair of leaves and subsequent two pairs), with the plant height at that stage of about 1 cm, and the other with inflorescences forming after four or five pairs of leaves and the plant reaching the height from 10 to 25 cm. The lower-forming inflorescences at the start of the fruit set would bend down to the soil, depositing the ripe fruit on the ground, while the higher-forming inflorescences would bend down noticeably but, because of the main stem height, would be unable to touch the soil surface. Inflorescences with the fruit not set (a rare phenomenon) stay upright. Later observations of plants growing on moss-covered ground showed that the capsules are actually buried in the soft substrate (Fig. 2G).

#### Etymology.

 The specific name refers to the sometimes repeated bending of its infructescence branches to the ground, figuratively evoking an image of the etiquette of genuflexion.

#### Preliminary conservation status.

 The species is known from only a handful of collections from two restricted populations in a non-protected area (private land), and should therefore be assessed as Data Deficient for EOO and AOO, following IUCN (2001)'s criteria.

#### Phenology.

 The species has been found flowering and fruiting from March to November during the local rainy season. It takes about 3–4 weeks from anthesis to fruit maturity. Living plants have not been observed from December to early March.

#### Specimens examined.

** Brazil: **Bahia: Entre Rios: Fazenda Rio do Negro, Residual stands of the Atlantic Forest. Restinga-type forest of the Rio do Negro valley, ca. 15 km southeast of Entre Rios, Atlantic forest, 12°01'S, 38°02'W, 150 m (topotypes), 3 June 2009, *A.V. Popovkin 598* (HUEFS); ibid., 10 June 2009, *A.V. Popovkin 602* (CHRB, NY); ibid., 15 July 2009, *A.V. Popovkin 602A *(CHRB, NY); ibid., 31 July 2009, *A.V. Popovkin 617* (HUEFS); ibid., 27 May 2010, *A.V. Popovkin 703* (HUEFS); ibid., 4 Sep 2010, *A.V. Popovkin 744* (HUEFS); ibid., 18 January 2011, *A.V. Popovkin 825 *(HUEFS); ibid., 8 June 2011, *A.V. Popovkin & J.C. Mendes 885* (HUEFS). Bahia: Entre Rios: Imbé, Atlantic forest, 12°05'S, 38°W, 135 m: 1 October 2010, *A.V. Popovkin & J.C. Mendes 758 *(HUEFS); 1 June 2011, *A.V. Popovkin & J.C. Mendes 878 *(HUEFS); 8 June 2011, *A.V. Popovkin & J.C. Mendes 885 *(HUEFS); 17 August 2011, *A.V. Popovkin & J.C. Mendes 913 *(HUEFS).

## Methods

### Morphological and molecular studies.

Photographs in the field and of cultivated material were made using a Panasonic DMC-ZS3 camera. Pressed and dried herbarium material of *Spigelia genuflexa* were observed, measured and photographed using a Stemi-2000 Zeiss dissecting microscope with a mounted digital Canon camera.Measurements were made using a caliper or using a graded and calibrated eye piece in a dissecting scope.

Several species concepts were utilized to identify and define this particular species, which is in line with previous species concepts used in this group (Gould 1999; Gould and Jansen 1999). Overall morphology provides a unique combination of characters supporting a new species based on the traditional morphological species concept. Additionally, the phylogenetic species concept also supports the status accorded here by the species' isolated evolutionary position and its autapomorphies in the phylogenetic analyses.

Sequences from the internal transcribed spacer (ITS) of nuclear ribosomal DNA were used to reconstruct a phylogenetic tree of 15 species of *Spigelia*, including the new species, and several outgroups. The complete methods for the phylogenetic analysis are presented in Appendix I.

## Results

**Figure 3.**
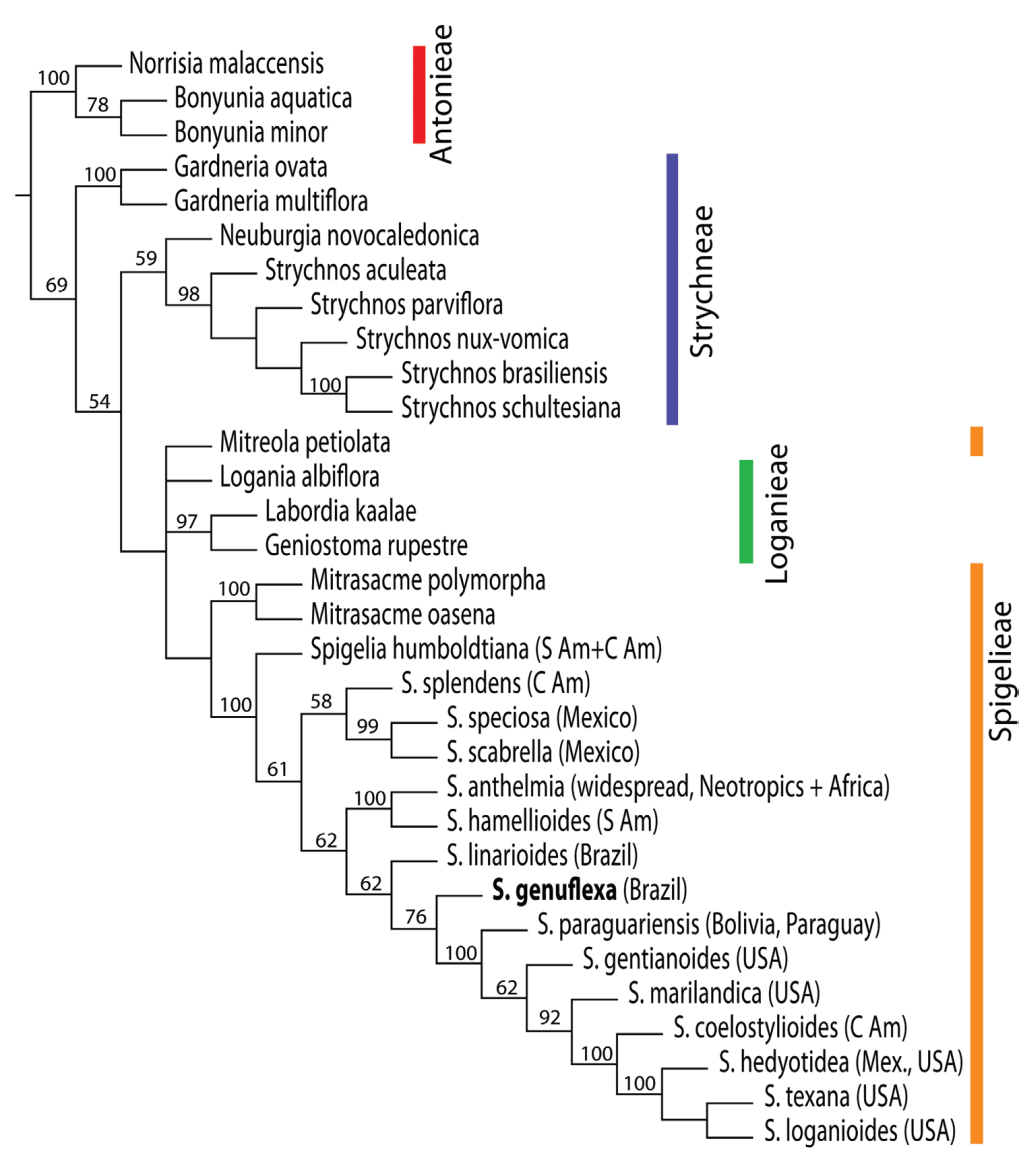
Strict consensus tree derived from molecular data (ITS and coded gaps) showing phylogenetic relationships of *Spigelia* and outgroups within Loganiaceae (tribal classification according to Leeuwenberg and Leenhouts 1980). Numbers above branches indicate % jackknife support above 50%.

**Figure 4.**
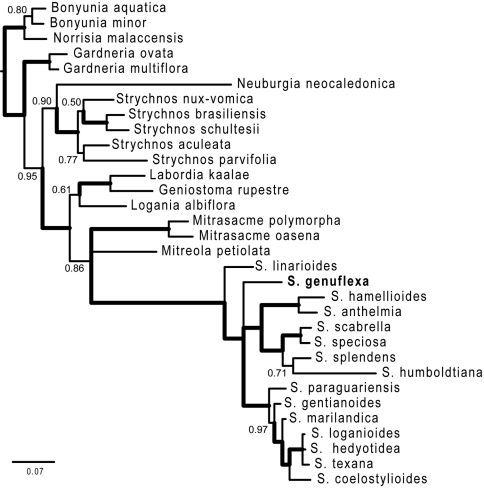
50% majority rule consensus tree from the Bayesian analyses. Numbers are clade posterior probability (pp) values; thickened branches indicate pp = 1.00. The genus name *Spigelia* is abbreviated to the first letter.

Complete phylogenetic results are presented in Appendix I. Thus far, we have been able to include only one other Brazilian species in the phylogenetic analysis, therefore our results are to be viewed as preliminary but having a bearing on the status of the new species. ITS sequences confirm the position of *Spigelia genuflexa* within the genus *Spigelia* relative to multiple outgroups in Loganiaceae. The strict consensus of two most parsimonious trees is shown in Figure 3. In this tree *Spigelia genuflexa* is placed as sister to a clade containing five northern warm-temperate taxa and two tropical taxa. The only other strictly Brazilian species included in the analysis, *Spigelia linarioides* DC., is positioned on the node just below *Spigelia genuflexa*. Below the branch with *Spigelia linariodes* is a clade formed by the two widespread species, *Spigelia anthelmia* and *Spigelia hamellioides*, and below this a clade of three Mexican species. *Spigelia humboldtiana*, a widespread species from central South America to southern Mexico, is most basal in *Spigelia*. Figure 4 shows the results of the Bayesian analysis, which differ from the parsimony results primarily in the positions of *Spigelia linarioides* and *Spigelia humboldtiana*: *Spigelia linariodes* from Brazil is on a basal branch outside of all other *Spigelia* species, and *Spigelia humboldtiana* is sister to a Mexican species, *Spigelia splendens* H. Wendl. ex Hook. In the Bayesian analysisthe position of *Spigelia genuflexa* remains unresolved, but phylogenetically distinct.

## Discussion

**Morphology.** Morphologically, *Spigelia genuflexa* displays a mosaic of traits similar to other species, as well as several unique characteristics, including geocarpy. Its leaves are densely covered with short, simple, slightly hooked papillae-like hairs, a trait that appears to be unique within the genus. Its small, white, funnelform flowers with included stamens (vs. long-tubular or campanulate, brightly colored flowers, with exserted stamens) make it similar to *Spigelia flemmingiana* (not included in the phylogeny), *Spigelia anthelmia*, *Spigelia hamellioides* (syn. *Spigelia multispica* Steud.), and many others. Other traits shared with the latter three species are the annual lifespan, the warty (vs. smooth) capsule and the persistent fruit base with pointed tips (vs. rounded or emarginate tips). Like *Spigelia anthelmia*, *Spigelia genuflexa* has a persistent style segment on the capsule that is short and stout (vs. long and threadlike). Like *Spigelia hamellioides*, *Spigelia genuflexa* has pedunculate inflorescences (vs. sessile). *Spigelia genuflexa* is distinctly different from other species occurring in the Atlantic coastal forest biome of Bahia, including *Spigelia anthelmia*, *Spigelia blanchetiana* A.DC., *Spigelia flemmingiana* Cham. & Schltdl., *Spigelia glabrata* Mart., *Spigelia laurina* Cham. & Schltdl., *Spigelia linarioides* (in the phylogeny), *Spigelia schlechtendaliana* Mart., *Spigelia spartioides* Cham., and *Spigelia tetraptera* Taub. ex L.B. Sm.. Determining the definitive relationships of *Spigelia genuflexa* within *Spigelia* will undoubtedly depend on the future inclusion of many additional species in a phylogenetic analysis.

**Phylogeny.** Very little phylogenetic work has been published in *Spigelia*, despite it being a relatively large genus with interesting Neotropical distribution and variable morphology linked to ecological traits, such as life span, pollination syndromes, and weediness (but see Gould 1997). This preliminary study is limited in its taxon selection, but it includes species from five of the seven described sections (Torrey and Gray 1839, Progel 1868, Bravo 1971) and all geographical regions except the Caribbean (including North America, Mexico, Central America and South America), and is a first approximation of relationships of some major clades.

The main goal with our phylogenetic analysis, however, was to place *Spigelia genuflexa* in a taxonomic neighborhood within its genus and the Loganiaceae. It is clearly supported as included in *Spigelia*, and it is also clearly not very close to any other *Spigelia* species we have sequenced so far, based on sequence similarity or position in the cladograms. In fact, we cannot say with any certainty where its affinities lie since it is not definitively grouped with any other species. There is low jacknife support in the parsimony analysis for its inclusion in a clade with *Spigelia paraguariensis* Chodat and more northern species. It does not group with any member of section *Anthelmiae* Progel (*Spigelia anthelmia*, *Spigelia hamellioides* and *Spigelia humboldtiana*), with which it shares morphological similarities, although this section is not monophyletic in any analyses. It also does not group with the only other Brazilian species, *Spigelia linarioides*, which is understandable, considering their quite different vegetative morphologies.

Like section *Anthelmiae*, sections *Graciles* Progel, *Stenophyllae* Progel and *Speciosae* Progel are not monophyletic in any analyses. This is not surprising, given the superficial nature of Progel's (1868) classification, based on a small number of morphological characters. Vegetative characters used by Progel, which may be highly plastic, include vestiture, stem shape and leaf venation. Floral characters he used, which may be related to pollinator selection, include corolla shape and exsertion of anthers and stigma. In the future, mapping morphological characters onto a better resolved molecular phylogeny might give some insight into character evolution in *Spigelia*. The one infrageneric classification that holds up in all analyses is the North American section *Coelostylis *(Torr. & A. Gray) Fern. Casas, the members of which (*Spigelia texana *(Torr. & A. Gray) A. DC., *Spigelia loganioides *(Torr. & A. Gray) A. DC., *Spigelia hedyotidea* A. DC.) form a strongly supported clade. According to our trees, the more tropical *Spigelia coelostylioides* K.R. Gould is closely related to this group, as hypothesized earlier (Gould 1999).

The sister group to *Spigelia* is shown to be other members of tribe Spigelieae sensu Leeuwenberg and Leenhouts (1980), with strong support in the Bayesian analysis (1.00 pp). *Mitrasacme* Labill. is sister to *Spigelia* in the parsimony tree, but with less than 50% jacknife support. *Mitrasacme* and *Mitreola* L. share a clade with *Spigelia* in the Bayesian analysis (0.86 pp), though which of the former is closer to *Spigelia* remains unresolved. In the parsimony tree, *Mitreola*'s position within Loganiaceae is also unresolved.

**Dwarfism in ephemeral annuals. **Dwarfism is also seen in two other *Spigelia* species, *Spigelia pygmaea* D.N. Gibson and *Spigelia polystachya* Klotzsch, both of which are annuals and are characteristically shorter than other *Spigelia* species. We have not yet been able to include either of these in the phylogenetic analysis. *Spigelia pygmaea* is known only from Chiapas, Mexico, and Guatemala, growing in generally dry habitats, including dry, deciduous forest, pine forest, and savanna. Like *Spigelia genuflexa*, it has warty-papillose capsules. *Spigelia polystachya* grows in seasonally flooded fields and mud flats from southern lowland Mexico south to Bahia and Goiás, Brazil, and appears to flower year-round. Since the only collection of *Spigelia genuflexa* of much taller size (to 25 cm) was made in the multi-layer leaf litter of the *tabuleiro* forest, the dwarfism of the initially collected plants could be strongly influenced by the differences in their respective micro-habitats, with the dwarfed plants found in a rather inhospitable bare sandy soil environment, with very little leaf litter. Such size plasticity is not uncommon in plants; however, even the larger specimens of *Spigelia genuflexa* represent plants of a small stature.

**Geocarpy. **There are several adaptive advantages of geocarpy for plants growing in variable, heterogeneous, or ephemeral environments, such as the retention of offspring in advantageous microhabitats, protection of seeds from environmental extremes, fire, and predators. The depositor-style geocarpy, in Hylander's terminology (Hylander 1929), is also occasionally found in some species of other plant families, e.g. Begoniaceae, Brassicaceae, Campanulaceae, Caryophyllaceae, Fabaceae, Hypoxidaceae, Myrsinaceae, Ranunculaceae, Rosaceae, Scrophulariaceae (Rawitscher 1937; Agnew and Hedberg 1968–69; Van der Pijl 1982; Cheplick 1987; Bruhl 1994; Barker 2005).

## Conclusion

*Spigelia genuflexa* is a new and unique species, with geocarpic fruits, the first known case of geocarpy in the Loganiaceae. It is not surprising it has not been detected earlier, given its diminutive stature and high biodiversity in the area. Northeastern Brazil contains the greatest number of known *Spigelia* species, most of which have been little studied. To better understand the taxonomic and distributional ranges of *Spigelia* species in Brazil, the threats to their survival, and their relationships and evolution, and last but not least, to get a better estimate of their actual number, a revision of Brazilian species is greatly needed.

## Supplementary Material

XML Treatment for Spigelia genuflexa

## Hyperlinked Genbank accession numbers:

### Hyperlinked Genbank accession numbers:

**Table:**

*Bonyunia aquatica*	JF937926	http://www.ncbi.nlm.nih.gov/nuccore/JF937926
*Bonyunia minor*	JF937927	http://www.ncbi.nlm.nih.gov/nuccore/JF937927
*Gardneria multiflora*	JF937929	http://www.ncbi.nlm.nih.gov/nuccore/JF937929
*Gardneria ovata*	JF937930	http://www.ncbi.nlm.nih.gov/nuccore/JF937930
*Geniostoma rupestre*	DQ499095	http://www.ncbi.nlm.nih.gov/nuccore/DQ499095
*Labordia kaalae*	JF937931	http://www.ncbi.nlm.nih.gov/nuccore/JF937931
*Logania albiflora*	DQ358879	http://www.ncbi.nlm.nih.gov/nuccore/DQ358879
*Mitrasacme oasena*	JF937932	http://www.ncbi.nlm.nih.gov/nuccore/JF937932
*Mitrasacme polymorpha*	JF937933	http://www.ncbi.nlm.nih.gov/nuccore/JF937933
*Mitreola petiolata*	AF054635	http://www.ncbi.nlm.nih.gov/nuccore/AF054635
*Neuburgia novocaledonica*	JF937935	http://www.ncbi.nlm.nih.gov/nuccore/JF937935
*Norrisia malaccensis*	JF937936	http://www.ncbi.nlm.nih.gov/nuccore/JF937936
*Spigelia anthelmia*	JF937937	http://www.ncbi.nlm.nih.gov/nuccore/JF937937
*Spigelia coelostylioides*	AF177992	http://www.ncbi.nlm.nih.gov/nuccore/AF177992
*Spigelia gentianoides*	JN005877	http://www.ncbi.nlm.nih.gov/nuccore/JN005877
*Spigelia genuflexa *	JN005878	http://www.ncbi.nlm.nih.gov/nuccore/JN005878
*Spigelia hamellioides*	JN005879	http://www.ncbi.nlm.nih.gov/nuccore/JN005879
*Spigelia hedyotidea*	AF178008	http://www.ncbi.nlm.nih.gov/nuccore/AF178008
*Spigelia humboldtiana*	JN005881	http://www.ncbi.nlm.nih.gov/nuccore/JN005881
*Spigelia linarioides*	JN005880	http://www.ncbi.nlm.nih.gov/nuccore/JN005880
*Spigelia loganioides*	AF178000	http://www.ncbi.nlm.nih.gov/nuccore/AF178000
*Spigelia marilandica*	AF177991	http://www.ncbi.nlm.nih.gov/nuccore/AF177991
*Spigelia paraguariensis*	JN005882	http://www.ncbi.nlm.nih.gov/nuccore/JN005882
*Spigelia scabrella*	JN005885	http://www.ncbi.nlm.nih.gov/nuccore/JN005885
*Spigelia speciosa*	JN005884	http://www.ncbi.nlm.nih.gov/nuccore/JN005884
*Spigelia splendens*	JN005883	http://www.ncbi.nlm.nih.gov/nuccore/JN005883
*Spigelia texana*	AF178006	http://www.ncbi.nlm.nih.gov/nuccore/AF178006
*Strychnos aculeata*	JF937940	http://www.ncbi.nlm.nih.gov/nuccore/JF937940
*Strychnos brasiliensis*	JF937956	http://www.ncbi.nlm.nih.gov/nuccore/JF937956
*Strychnos nux-vomica*	JF938015	http://www.ncbi.nlm.nih.gov/nuccore/JF938015
*Strychnos parvifolia*	JF938021	http://www.ncbi.nlm.nih.gov/nuccore/JF938021
*Strychnos schultesiana*	JF938036	http://www.ncbi.nlm.nih.gov/nuccore/JF938036

## Appendix I. Molecular phylogenetic methods and results

**Molecular studies. **Total genomic DNA extraction of *Spigelia genuflexa* was done by grinding one leaf from a NY herbarium specimen with sterile glass pestle in a mortar, and then processed using the DNAeasy Plant Mini Kit (Qiagen), according to the manufacturer's instructions.

The nuclear ITS rDNA region was amplified using the primers 5'-AACAAGGTTTCCGTAGGTGA-3' (modified from Baldwin 1992) and 5'-GCTACGTTCTTCATCGATGC-3' (White et al. 1990) to ITS1 and 5'-GCATCGATGAAGAACGTAGC-3' (White et al. 1990) and 5'-TATGCTTAAAYTCAGCGGGT (modified from Baldwin 1992) to ITS2. PCR amplifications were performed according to Frasier et al. (2008) and conducted on an Applied Biosystems GeneAmp System 9700, using the program: 97oC for 1 min, followed by 35 cycles of 95oC for 1 min, 53oC for 1 min, and 68oC for 2 min, ending with a final extension of 72oC for 4 min. For visualization, PCR products were run on 1% agarose gel. PCR products that resulted in single bands were cleaned using ExoSAP-IT® (USB cat.# 78201), following manufacturer's specifications, and then submitted to Genewiz Inc. for sequencing.

Additional ITS sequences from other *Spigelia* species were provided by co-author K. Mathews (Gould 1997; see methods therein for those sequences) and from Genbank. Sequences of other Loganiaceae species were obtained from C. Frasier (Frasier 2008). For a list of included species, classification, Genbank numbers and vouchers, see Table 1.

**Table 1.** Material for phylogenetic analysis using ITS sequences, with voucher information, Genbank accession number, and tribal classification according to Leeuwenberg and Leenhouts (1980). Herbarium abbreviations according to Index Herbariorum.

Species	Tribal classification	Infrageneric classification of Spigelia, if applicable	Genbank accession number	Voucher or publication
*Bonyunia aquatica*	Antonieae		JF937926	Berry et al. 5771 (NY)
*Bonyunia minor*	Antonieae		JF937927	Berry & Brako 5522 (NY)
*Gardneria multiflora*	Strychneae		JF937929	Ceming 9611186 (MO)
*Gardneria ovata*	Strychneae		JF937930	Klackenberg & Lundin 214 (NY)
*Geniostoma rupestre*	Loganieae		DQ499095	Wright et al. (2006)
*Labordia kaalae*	Loganieae		JF937931	Motley 1203 (BISH)
*Logania albiflora*	Loganieae		DQ358879	Hubbard 4198 (G)
*Mitrasacme oasena*	Spigelieae		JF937932	Forste et al. PIF24800 (NY)
*Mitrasacme polymorpha*	Spigelieae		JF937933	Anonymous 20495 (NY)
*Mitreola petiolata*	Spigelieae		AF054635	Gould 150 (TEX/LL)
*Neuburgia novocaledonica*	Strychneae		JF937935	Struwe 1301 (NY)
*Norrisia malaccensis*	Antonieae		JF937936	Stone 14107 (HUH)
*Spigelia anthelmia*	Spigelieae	Anthelmiae	JF937937	Worthington 21205 (NY)
*Spigelia coelostylioides*	Spigelieae		AF177992	Gould 139 (TEX/LL)
*Spigelia gentianoides*	Spigelieae		JN005877	Bok Tower Gardens Rare Plant Collection, Lake Wales Florida (living collection)
*Spigelia genuflexa *	Spigelieae		JN005878	Popovkin 602 (NY)
*Spigelia hamellioides*	Spigelieae	Anthelmiae	JN005879	Gould 7 (TEX/LL)
*Spigelia hedyotidea*	Spigelieae	Coelostylis	AF178008	Gould 103 (TEX/LL)
*Spigelia humboldtiana*	Spigelieae	Anthelmiae	JN005881	Gould 162 (TEX/LL)
*Spigelia linarioides*	Spigelieae	Graciles	JN005880	Taylor et al. 1508 (K)
*Spigelia loganioides*	Spigelieae	Coelostylis	AF178000	Goldman 433 (TEX/LL)
*Spigelia marilandica*	Spigelieae	Graciles	AF177991	Gould 163 (TEX/LL)
*Spigelia paraguariensis*	Spigelieae	Stenophyllae	JN005882	Zardini & Velasquez 27462 (G)
*Spigelia scabrella*	Spigelieae	Stenophyllae	JN005885	Williams 9565 (TEX/LL)
*Spigelia speciosa*	Spigelieae	Speciosae	JN005884	Gould 136 (TEX/LL)
*Spigelia splendens*	Spigelieae	Speciosae	JN005883	Panero 5758 (TEX/LL)
*Spigelia texana*	Spigelieae	Coelostylis	AF178006	Gould 135 (TEX/LL)
*Strychnos aculeata*	Strychneae		JF937940	Merello 1338 (MO)
*Strychnos brasiliensis*	Strychneae		JF937956	Medri etal. 446 (NY)
*Strychnos nux-vomica*	Strychneae		JF938015	Maxwell 90–622 (MO)
*Strychnos parvifolia*	Strychneae		JF938021	Rodal et al. 502 (MO)
*Strychnos schultesiana*	Strychneae		JF938036	Liesner & Gonzalez 9170 (MO)

The *Spigelia genuflexa* sequence was edited and assembled using Sequencher ver. 4.10 (GeneCodes). The sequences were aligned using MUSCLE ver. 3.7 (Edgar 2004, 2010), with subsequent modification of the alignment in SEQUENCHER v. 4.10 (Gene Codes) to optimize gap alignments. The aligned ITS matrix included 32 taxa and 655 aligned nucleotides and 42% of the sites were phylogenetically informative (Appendix II). Indel characters (gaps) were coded using SEQSTATE ver. 1.4.1 (Müller 2005) using simple gap coding (Simmons and Ochoterena 2000), which lead to the addition of 114 binary gap characters (Appendix III).

**Phylogenetic analysis.** The phylogenetic data matrices were analyzed using maximum parsimony using NONA (Goloboff 2002), spawned via WINCLADA (Nixon 2002), and the following settings: unconstrained heuristic search, 1000 max trees, 500 replicates, 5 starting trees per replicate, multiple TBR + TBR. Jackknife analysis was also performed with NONA and WinClada, using 1000 replicates, 10 search reps per replicate, 5 starting trees per replicate, don't do max TBR, save consensus, and maxtrees = 1000. Two matrices were analyzed: 1) ITS only, and 2) ITS with gaps coded. Trees were rooted with members of tribe Antoniaeae, including *Norrisia* and *Bonyunia* spp., based on phylogenetic results in Frasier (2008).

For the second, Bayesian, analyses we chose the best fitting nucleotide substitution model (excluding the binary indel partition), using the Akaike information criterion (AIC) in JMODELTEST ver. 0.1.1 (Posada 2008). We analyzed the data using Bayesian inference in MRBAYES 3.1.2 (Huelsenbeck and Ronquist 2001). Data were partitioned into nucleotide and indel partitions (Appendix IV). The indel characters were assigned the binary model (nst=1, coding=variable), as recommended in the MrBayes manual. For the nucleotide characters, JMODELTEST chose TIM3 + G + I, a special case of GTR with two transversion rates, which is not available in MRBAYES. Instead, we assigned the GTR + G + I model (nst=6, rates=invgamma). All free parameters were estimated from the data using the default settings in MRBAYES and were unlinked for the two partitions. Two simultaneous analyses were run, each with four chains (one cold and three heated), for 1 million generations, sampling every hundredth generation. Convergence was evaluated by examining the standard deviation of split frequencies among runs (accepted value of <0.01). The resulting branch posterior probabilities and consensus topology were summarized using the sumt command, excluding trees from the initial 250,000 generations as burn-in.

**Results. **The parsimony analysis of ITS yielded only four most parsimonious trees, with 993 steps, consistency index (ci) = 0.59, and retention index (ri) = 0.68. When coded gap characters were included, two most parsimonious trees were found, with a length of 1348 steps, ri = 0.57, and ci = 0.73 (strict consensus tree shown in Figure 3, with jackknife support above branches). *Spigelia* is supported as monophyletic in both analyses, with a jackknife value of 99% in the ITS-only analysis. Placed as sister to *Spigelia* is *Mitrasacme*, but with low support (50%, ITS only; below 50%, ITS plus gaps).

The strict consensus trees from these two analyses are largely congruent, with a few exceptions. The results from ITS only differ from matrix 2 (ITS with coded gaps) by: 1) positioning a species of *Mitreola* as sister to *Logania*, and this clade as sister clade to *Labordia* and *Geniostoma*; 2) collapsing the node below *Strychnos parvifolia* within *Strychnos*; and 3) rearranging relationships among *Spigelia* species in the clade, including *Spigelia paraguariensis*. These three differences are nodes that are relatively poorly supported by jackknife analysis (up to 62% support) based on ITS only data.

The new species, *Spigelia genuflexa*, is placed as sister (76% support) to a clade containing five northern warm-temperate taxa (*Spigelia marilandica*, *Spigelia gentianoides*, *Spigelia texana*, *Spigelia hedyotidea*, *Spigelia loganioides*) in addition to two tropical taxa, *Spigelia paraguariensis* (Paraguay) and *Spigelia coelostylioides* (Chiapas, Mexico; Figure 3) in the parsimony results. The only other strictly Brazilian species included in the analysis, *Spigelia linarioides*, is positioned on the node right below *Spigelia genuflexa*. Below the branch with *Spigelia linariodes* is a clade formed by the two widespread species, *Spigelia anthelmia* and *Spigelia hamellioides*, and below this a clade of three Mexican species (*Spigelia scabrella*, *Spigelia speciosa*, *Spigelia splendens*). The species placed on the most basally positioned branch within *Spigelia* is *Spigelia humboldtiana*, a multi-stemmed, basally woody herb widespread from central South America to southern Mexico.

In the Bayesian analysis, *Mitreola* and *Mitrasacme* are in an unresolved sister clade to *Spigelia* (0.86 posterior probability [pp]), and *Spigelia* is monophyletic (1.00 pp; Figure 4). Within *Spigelia*, the Bayesian results differ from the parsimony results primarily in the positions of *Spigelia linarioides* and *Spigelia humboldtiana*: *Spigelia linariodes* from Brazil is on a basal branch outside of all other *Spigelia* species (1.00 pp), and *Spigelia humboldtiana* is sister to a Mexican species, *Spigelia splendens* (0.71 pp). *Spigelia genuflexa*'s position remains unresolved, but phylogenetically distinct, in the Bayesian analysis. We experimented with setting priors in the Bayesian inference by fixing the substitution rates and nucleotide frequencies to the values in the Q-matrices output by jModelTest (results not shown). This corresponded to the more parameter-rich TIM3 model. The resulting consensus tree placed *Spigelia genuflexa *as sister to the *Spigelia paraguariensis* + North American species clade (the same position as in the parsimony analyses), albeit with a low posterior probability (0.55). Other relationships within *Spigelia* did not change.

## Appendix II

DNA alignment of ITS from Loganiaceae taxa, especially *Spigelia*, shown in Fasta format. (doi: 10.3897/phytokeys.6.1654.app2)

Copyright notice: This dataset is made available under the Open Database License (http://opendatacommons.org/licenses/odbl/1.0/). The Open Database License (ODbL) is a license agreement intended to allow users to freely share, modify, and use this Dataset while maintaining this same freedom for others, provided that the original source and author(s) are credited.

**Citation:** Popovkin AV, Mathews KG, Santos JCM, Molina MC, Struwe L (2011) *Spigelia genuflexa* (Loganiaceae), a new geocarpic species from the Atlantic forest of northeastern Bahia, Brazil. PhytoKeys 6: 47–65. doi: 10.3897/phytokeys.6.1654.app2

## Appendix III

Alignment of ITS from Loganiaceae taxa, especially *Spigelia*, including coded gaps, Nexus format. (doi: 10.3897/phytokeys.6.1654.app3)

Copyright notice: This dataset is made available under the Open Database License (http://opendatacommons.org/licenses/odbl/1.0/). The Open Database License (ODbL) is a license agreement intended to allow users to freely share, modify, and use this Dataset while maintaining this same freedom for others, provided that the original source and author(s) are credited.

**Citation:** Popovkin AV, Mathews KG, Santos JCM, Molina MC, Struwe L (2011) *Spigelia genuflexa* (Loganiaceae), a new geocarpic species from the Atlantic forest of northeastern Bahia, Brazil. PhytoKeys 6: 47–65. doi: 10.3897/phytokeys.6.1654.app3

## Appendix IV

Bayesian run-file of ITS plus coded gaps from Loganiaceae taxa, especially *Spigelia*, Nexus format. (doi: 10.3897/phytokeys.6.1654.app4)

Copyright notice: This dataset is made available under the Open Database License (http://opendatacommons.org/licenses/odbl/1.0/). The Open Database License (ODbL) is a license agreement intended to allow users to freely share, modify, and use this Dataset while maintaining this same freedom for others, provided that the original source and author(s) are credited.

**Citation:** Popovkin AV, Mathews KG, Santos JCM, Molina MC, Struwe L (2011) *Spigelia genuflexa* (Loganiaceae), a new geocarpic species from the Atlantic forest of northeastern Bahia, Brazil. PhytoKeys 6: 47–65. doi: 10.3897/phytokeys.6.1654.app4
